# Supplementary material for: The interactome of intact mitochondria by cross-linking mass spectrometry provides evidence for coexisting respiratory supercomplexes
Source: Mol Cell Proteomics. 2017 Dec 8;17(2):216–32. doi: 10.1074/mcp.RA117.000470 (PMC5795388; doi:10.1074/mcp.RA117.000470)
Supplement: Supplemental Data [file supp_RA117.000470_133922_0_supp_23978_fzffwf.zip › spectra_annotation/mito_spectra_annotation/433-1-6-1-1-1.pdf]

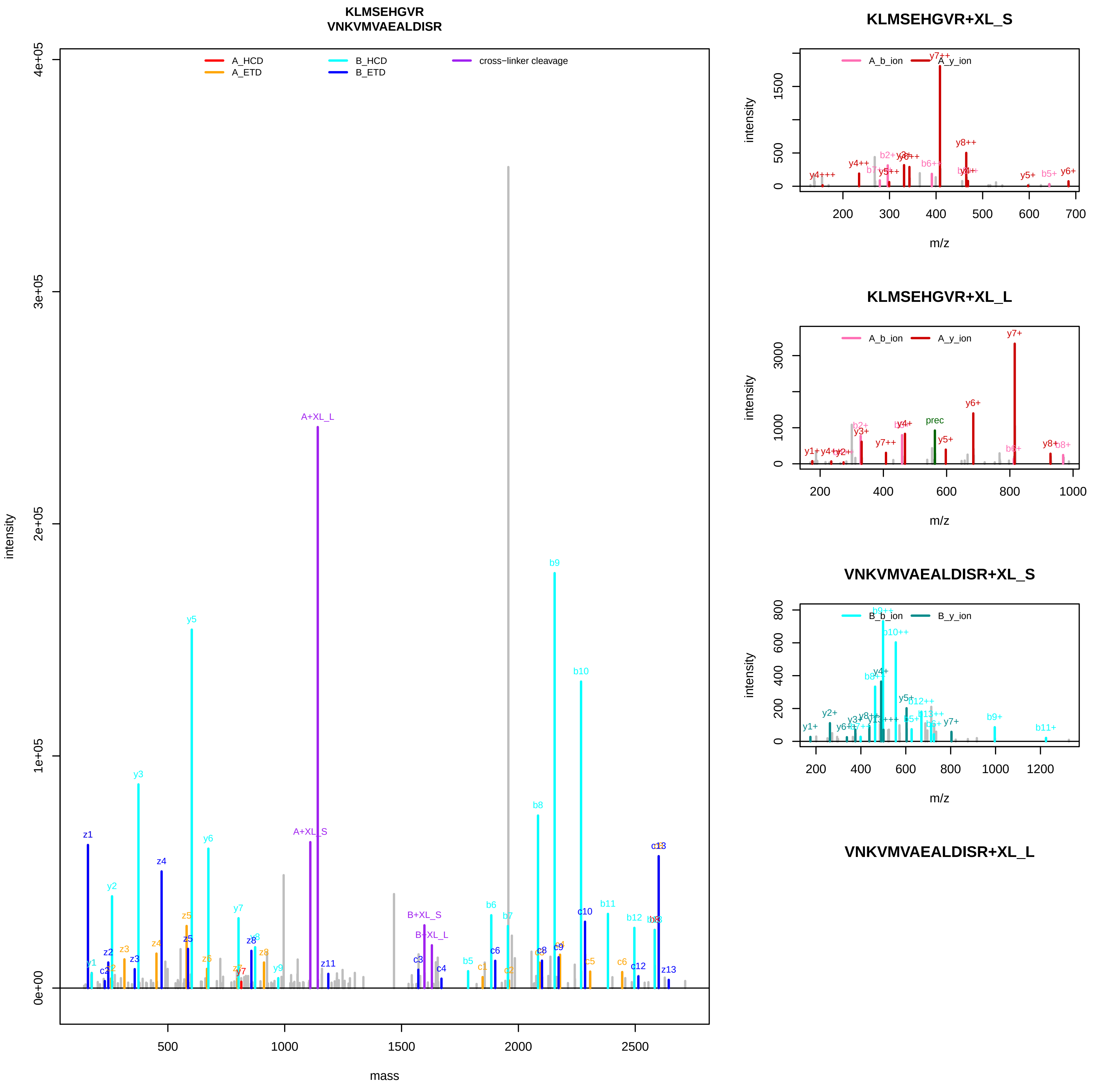
KLMSEHGVR
VNKVMVAEALDISR
A_HCD
A_ETD
B_HCD
B_ETD
cross−linker cleavage
intensity
mass
0e+00
1e+05
2e+05
3e+05
4e+05
500
1000
1500
2000
2500
A+XL_L
A+XL_S
B+XL_S
B+XL_L
y1
y2
y3
y5
y6
y7
y9
z1
z2
z3
z4
z5
z8
z11
z13
b5
b6
b7
b8
b9
b10
b11
b12
c3
c4
c6
c8
c9
c10
c12
c13
KLMSEHGVR+XL_S
KLMSEHGVR+XL_L
VNKVMVAEALDISR+XL_S
VNKVMVAEALDISR+XL_L
A_b_ion
A_y_ion
B_b_ion
B_y_ion
m/z
intensity
y7++
y8++
y7+
y6+
prec
b9++
b10++
y4+
y5+
y2+
b9+
b11+
